# Publication Trends of Research on Polypoidal Choroidal Vasculopathy During 2001–2020: A 20-Year Bibliometric Study

**DOI:** 10.3389/fmed.2021.785126

**Published:** 2022-01-31

**Authors:** Yimin Wang, Minyue Xie, Min Zhang, Xiaohuan Zhao, Xinyue Zhu, Yuwei Wang, Yuhong Chen, Jieqiong Chen, Xiaodong Sun

**Affiliations:** ^1^Shanghai General Hospital, Shanghai Jiao Tong University School of Medicine, Shanghai, China; ^2^National Clinical Research Center for Eye Disease, Shanghai, China; ^3^Shanghai Key Laboratory of Ocular Fundus Diseases, Shanghai, China; ^4^Shanghai Engineering Center for Visual Science and Photomedicine, Shanghai, China; ^5^Beijing Tongren Hospital, Capital Medical University, Beijing, China

**Keywords:** polypoidal choroidal vasculopathy (PCV), age-related macular degeneration (AMD), bibliometric, anti-VEGF (vascular endothelial growth factor), pigment epithelial detachment

## Abstract

**Introduction:**

Polypoidal choroidal vasculopathy (PCV) is a special subtype of AMD, which is one of the leading threats to vision health worldwide. At this time, many aspects of PCV, from how it works to potential treatments, remain a mystery. In this study, we explored the frontier researches and revealed the study trends within the study of PCV.

**Methods:**

We collected all the publications in this field from 2001 to 2020, analyzed trends within them, and defined the contributions of various countries/regions, institutions, authors, and journals. Additionally, VOSviewer software was used to define the hot keywords in this field.

**Results:**

A total of 1,190 publications were ultimately examined; We found that PCV is becoming an increasingly relevant topic of research, and that Japan has contributed the most publications (428), the most citations (14,504 in total), and the highest H-index value (62) to the field. Our keywords analysis was classified into four clusters to show the hotspots within the study of PCV, namely mechanism-related, imaging-related, prognosis-related, and therapy-related topics. The average years in which the keywords appeared the most were also calculated, and we identified anti-VEGF therapy, anti-complement therapy and angiography as having been the main focus in recent years.

**Conclusions:**

These results helped clarify the comprehensive research progress that has been made as well as the future trends in the study of PCV, which can assist and guide future research.

## Background

Polypoidal choroidal vasculopathy (PCV) is characterized by orange nodules in the macula and is regarded as a special subtype of AMD (1). In contrast to the clinical manifestations of typical AMD, PCV patients usually show thicker choroid, serous, or hemorrhagic pigment epithelial detachments (PEDs) (2, 3). PCV is particularly prevalent in racially diverse populations; about 20–60% of Asian patients diagnosed with AMD were classified as PCV, according to indocyanine green angiography (ICGA), while only about 10% of European AMD patients were classified as PCV (4–6). These different clinical manifestations suggest the distinct pathological differences between PCV and AMD. In fact, the clinical nature and pathogenesis of PCV still remain controversial (1, 7). There are several extant difficulties in terms of the diagnosis of PCV as well. ICGA, an invasive examination, is currently the gold standard for PCV diagnosis (8), though its incommodious diagnostic criteria may lead to the underestimation of the overall prevalence of PCV (9). There are also several challenges with current PCV treatments. Both the LAPTOP study and the EVEREST study confirmed the better visual outcome of anti-VEGF monotherapy as compared to photodynamic therapy (PDT) (10, 11); however, anti-VEGF requires repeated injections and its effect on the polyp regression rate in patients (17–40%) is limited (1, 12).

Due to these issues, a comprehensive review of the extant publications in the field of PCV is urgently needed. Bibliometric analyses are powerful tools for addressing this problem as they can define the current scholarly development in a specific field (13–15) and guide its future research direction. In this study, we explored the frontier researches of and revealed the study trends within the study of PCV. We also predicted trends for the next few years, noting that increases in the amount of relevant, high-quality research are expected to lead to a deeper understanding of PCV.

## Results

### Contributions of Various Countries and Regions to Global Publications

**Figure 1:**
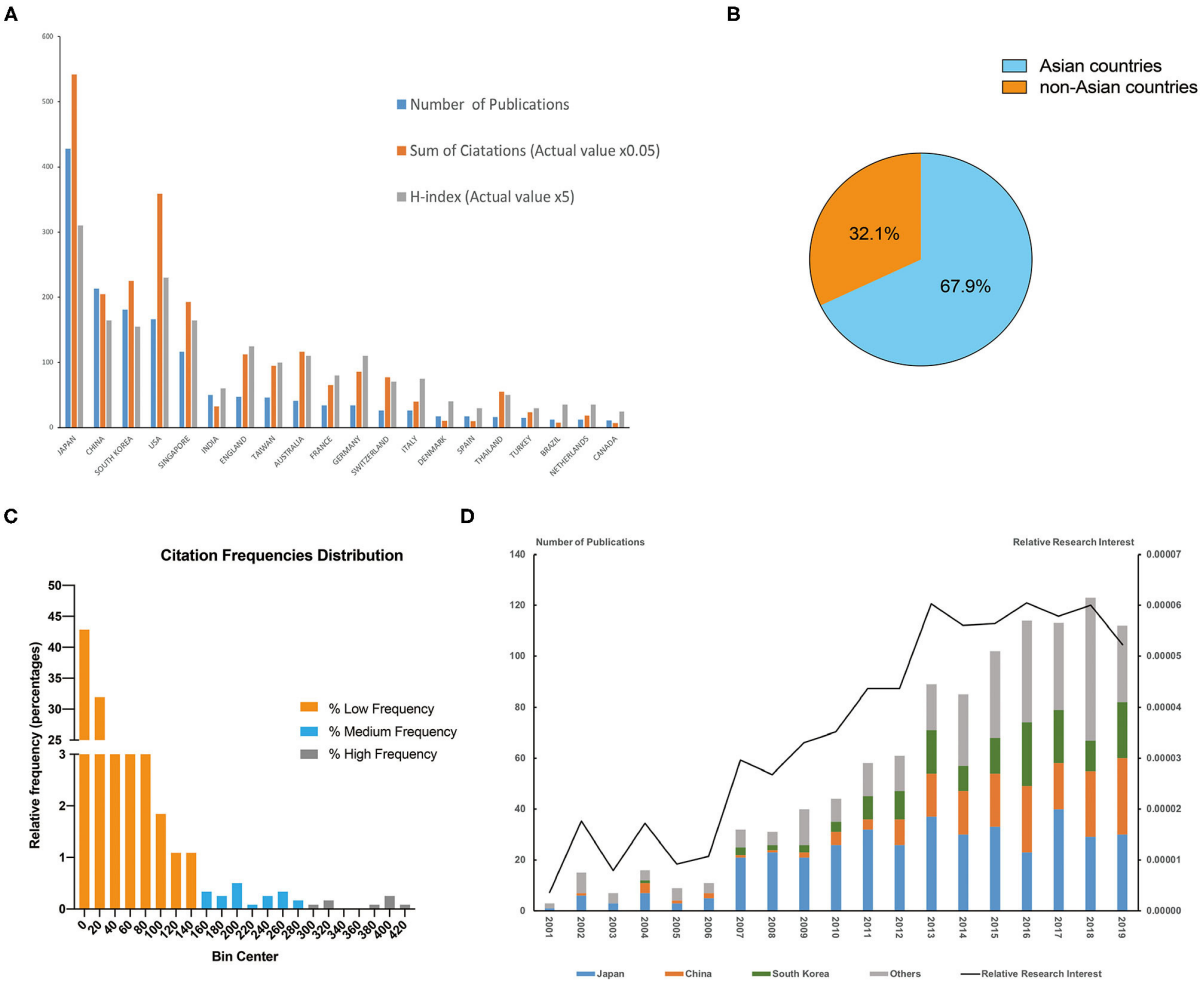
**(A)** top 20 countries (regions) in the PCV publications. The blue bar showed the number of publications, orange bar showed the sum of citations in total (actual value multiply by 0.05), gray bar showed the H-index (actual value multiply by 5). **(B)** The proportion of publication numbers of Asian countries/regions and non-Asian countries. **(C)** The distribution of total citation numbers; The publications were divided into three groups according to the total citation frequencies, including high frequency of citations (more than 280 citations), medium frequency (more than 140 citations and <280 citations) and low frequency (<140 citations). **(D)** The relative research interest (RRI) and proportion of Japan, China, South Korea and others of each year in the field of PCV. The left axis represented the number publications, while the right axis represented the relative research interest.

**Figure 2:**
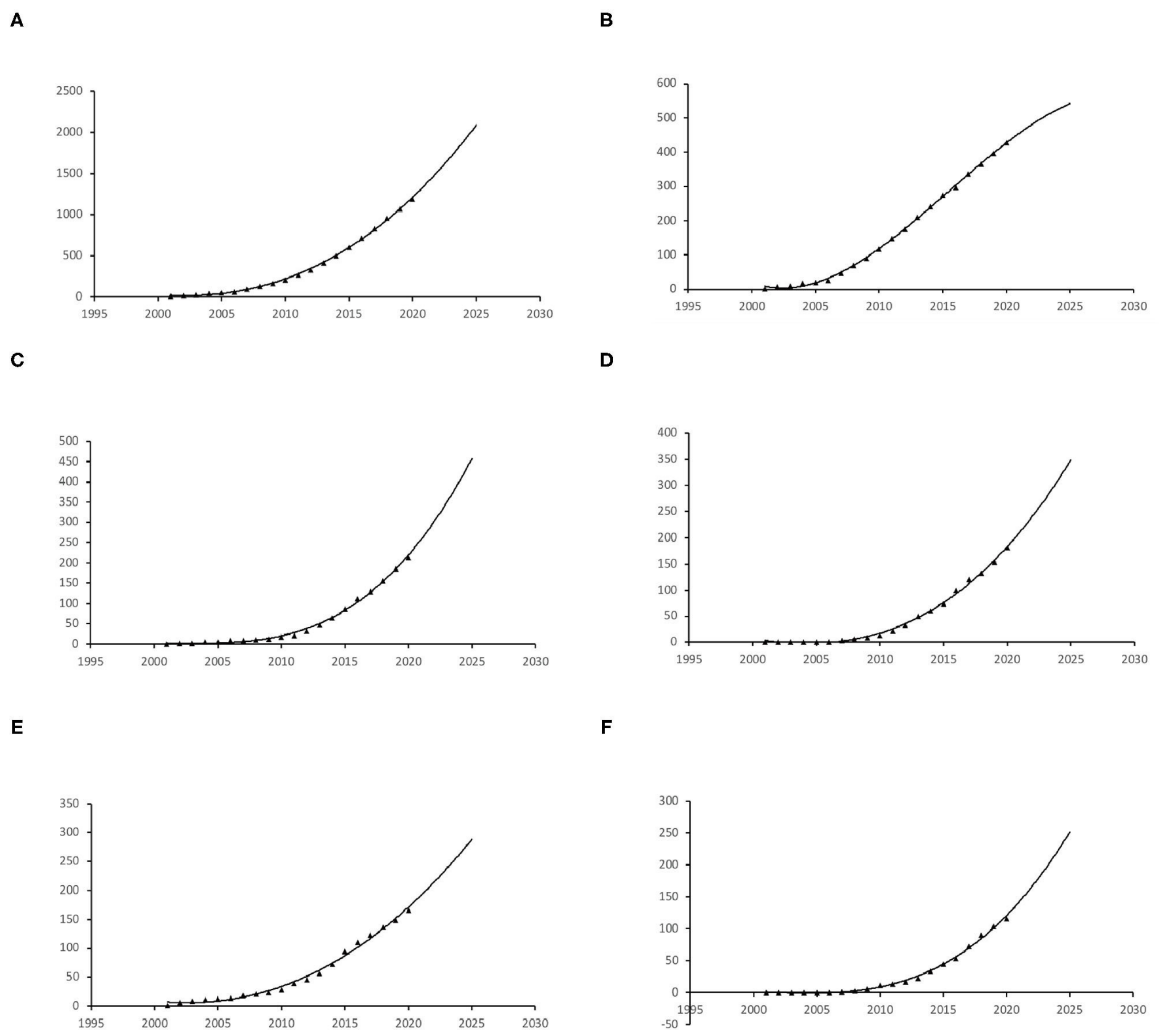
The publication growth trends of in the past 20 years and prediction curve. **(A)** Global, **(B)** Japan, **(C)** China, **(D)** South Korea, **(E)** USA, **(F)** Singapore.

The overall number of publications on PCV has maintained steady growth over the past 20 years (Figure 1D). From 2001 to 2020, Japan contributed to the most publications (428, 35.9%), followed by China (213, 17.9%), and South Korea (181, 15.2%) (Figure 1A). About one third of the publications were contributed by non-Asian countries, their studies were important part in this field (Figure 2B). In addition, half of the top 10 countries and regions with highest H-index were non-Asian countries/regions (Figure 1A). Japan also published the most papers per year from 2001 to 2020 (Figure 1D), peaking in 2017 with 40 publications. The publications of China and South Korea have substantial increase in this field. Additionally, relative research interest (RRI) also increased from <0.001% in 2001 to 0.005% in 2020, indicating that the global interest in this field has continued to increase over the past 20 years.

We also analyzed the co-occurrence of 22 countries and regions (Supplementary Figure 1); this analysis suggested five clusters, including 1. Japan, the Netherlands, Denmark, and Sweden; 2. China, Singapore, South Korea, India, Thailand, Malaysia, and Ireland; 3. The USA, France, England, Turkey, and Portugal; 4. Germany, Australia, Northern Ireland, and Spain; and 5. Switzerland and Greece.

### Citations and H-Index

WOS citation reports revealed that, of the 32,898 relevant citations since 2001, 17,707 citations without self-citations. Each paper was cited an average of 27.58 times and Japan contributed to the most citations (14,504, 10,838 without self-citations) and H-index (62) (Figure 1A) from 2001 to 2020. The United States ranked second in both citations and H-index (7,664 citations, 7,181 without self-citations, H-index 46) while China ranked third in terms of H-index (5,089 citations, 4,498 without self-citations, H-index 33).

The most cited publication has been cited for 411 times in total and the least cited publications have not been cited yet (Figure 1C). We have divided these publication into three groups according to the frequencies, including high frequency of citations (more than 280 citations), medium frequency (more than 140 citations and <280 citations) and low frequency (<140 citations). Most of the publications were in low frequency group, 27 papers were cited with a medium frequency and only 10 papers were cited with a medium frequency.

To further explore the distribution of citation number in each year, we supplemented the heatmap of each group (Supplementary Figure 3D). Every row in the heatmap represents a publication, the x axis means year, and the color represents the citation number. The time span of high frequency and medium frequency are longer than low frequency group. Besides, we also analyzed the distribution of publication year of each group (Supplementary Figure 3D). Most of the publications in low frequency group were published in recent years, and that could possibly due to less citations.

### Publication Trends and Predictions

**Figure 3:**
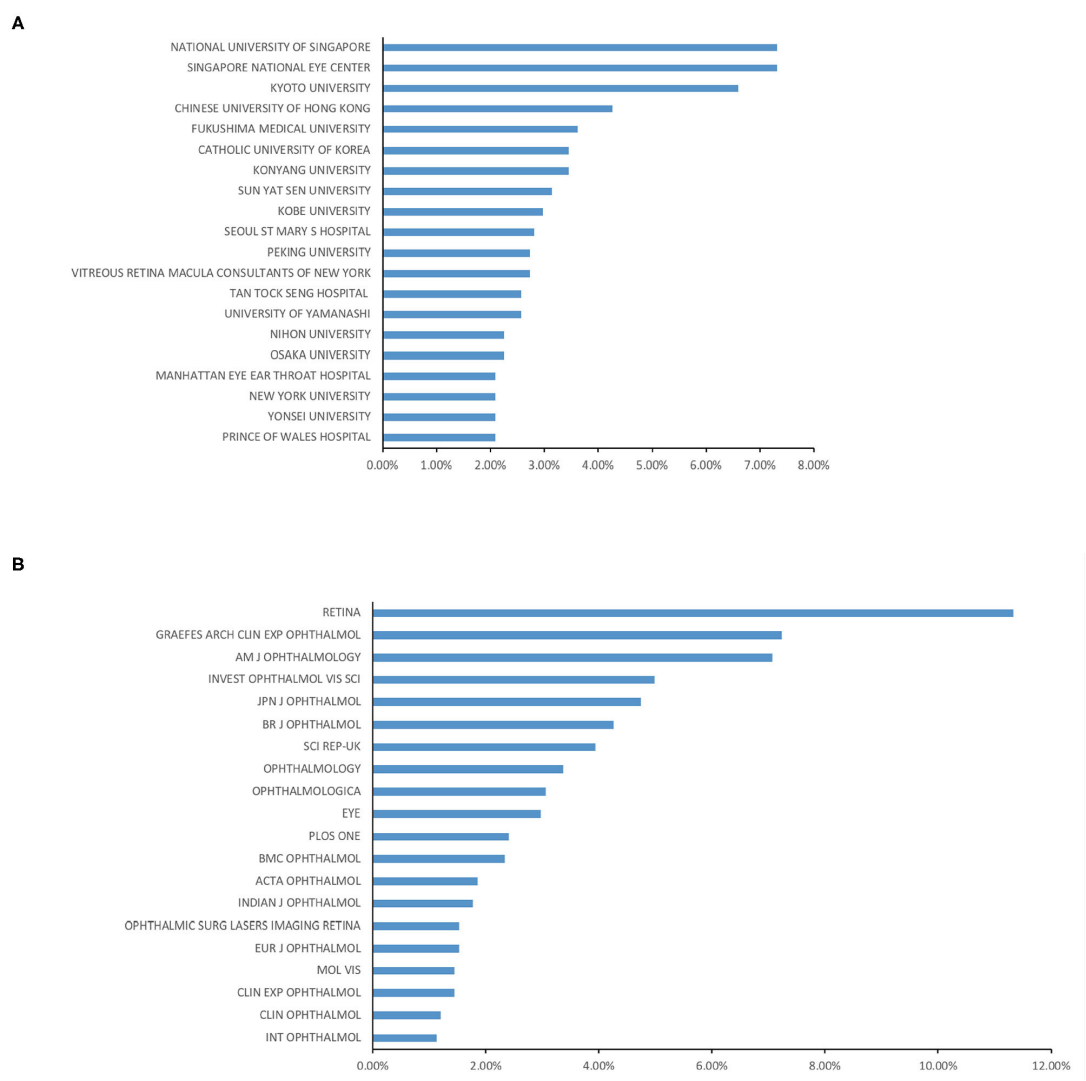
**(A)** Top 20 institutions all over the world ranked by the publication numbers. **(B)** Top 20 journals published most in this field.

The publication rate of papers on PCV has continued to increase over the past 20 years; predictions for the next 5 years show this increase continuing (Figure 2A). Japan is projected to maintain its leading position compared to the other countries and maintain steady growth (Figures 3B–F). However, China has shown the greatest steady increase in publication numbers since 2005 and is expected to produce more than 400 relevant publications over the next 5 years (Figure 2C).

### Institutions Publishing Research on PCV

We examined the top 20 institutions in this field and found that the National University of Singapore (91, 7.32%) and the Singapore National Eye Center (91, 7.32%) published the most papers on PCV in the past 20 years. Kyoto University ranked third (82, 6.59%), and the Chinese University of Hong Kong ranked fourth (53, 4.26%) (Figure 3A).

### Journals Publishing and High-Quality Publications Research on Polypoidal Choroidal Vasculopathy

More than 70% (903, 72.7%) of the papers on PCV were published in the same 20 journals, including *RETINA*, which published the most relevant publications (141). *GRAEFES ARCH CLIN EXP OPHTHALMOL* published the second-most with 90 publications (Figure 3B). In 2018, *Lancet* put forth a high-quality (with high impact factor) paper on PCV and age-related macular degeneration by Tien Y. Wong that comprehensively reviewed the mechanisms, clinical features, and treatment options of PCV (16).

The 10 papers with the most citations in total are displayed in Table 1; the most cited paper was published in *Ophthalmology*, a classic and authoritative ophthalmic periodical, and is called *Choroidal Thickness in Polypoidal Choroidal Vasculopathy and Exudative Age-related Macular Degeneration*. The corresponding author was Yun Taek Kim.

**Table 1:** The top 10 papers with the most citations in PCV research.

**Title**	**Corresponding authors**	**Journal**	**Publication year**	**Total citations**
Choroidal thickness in polypoidal choroidal vasculopathy and exudative age-related macular degeneration	Kim, Yun Taek	Ophthalmology	2011	411
Clinical characteristics of exudative age-related macular degeneration in Japanese patients	Saito, Kuniharu	American Journal of Ophthalmology	2007	392
Early-onset stroke and vasculopathy associated with mutations in ADA2	Aksentijevich, Ivona	Science	2014	392
Central serous chorioretinopathy: recent findings and new physiopathology hypothesis	Singh, Arun D.	Progress in Retinal and Eye Research	2015	392
EVEREST STUDY efficacy and safety of verteporfin photodynamic therapy in combination with ranibizumab or alone versus ranibizumab monotherapy in patients with symptomatic macular polypoidal choroidal vasculopathy	Lim, Tock H.	Retina-the Journal of Retinal and Vitreous Diseases	2012	382
Polypoidal choroidal vasculopathy—Incidence, demographic features, and clinical characteristics	Uyama, M	Archives of Ophthalmology	2003	321
Age-related macular degeneration	Wong, Tien Y.	Lancet	2018	313
Polypoidal choroidal vasculopathy: natural history	Matsumura, M	American Journal of Ophthalmology	2002	293
The prevalence of age-related macular degeneration in Asians A systematic review and meta-analysis	Wong, Tien Y.	Ophthalmology	2010	284
Aqueous humor levels of vascular endothelial growth factor and pigment epithelium-derived factor in polypoidal choroidal vasculopathy and choroidal neovascularization	Lam, DSC	American Journal of Ophthalmology	2006	282

### Authors Publishing Research on PCV

The top 10 authors in this field have published 532 papers, a number that accounts for about half of the total papers published (44.7%) in the past 20 years. The works of N. Yoshimura from Kyoto University were published the most in the past 20 years, with 72 papers and 2,451 citations (without self-citation). A. Tsujikawa, also from Kyoto University, ranked second with 67 publications and 2,151 citations (without self-citation) while K. Yamashiro from Otsu Red-Cross Hospital ranked third with 57 publications and 1,654 citations (without self-citation). All of the top 10 authors are from Asian countries/regions; six are from Japan (Table 2).

**Table 2:** Top 10 author who published most in this field.

**Author**	**Country**	**Affiliation**	**No. of publications**	**No. of citations**
Yoshimura N.	Japan	Kyoto University	72	2,451
Tsujikawa A	Japan	Kyoto University	67	2,151
Yamashiro K	Japan	Otsu Red-Cross Hospital	57	1,654
Wong TY	Singapore	National University of Singapore	56	2,561
Cheung CMG	Singapore	Singapore National Eye Center	47	1,532
Iida T	Japan	Tokyo Women's Medical University	43	1,891
Lai TYY	China	The Chinese University of Hong Kong	40	2,022
Tamura H	Japan	Kyoto University Graduate School of Medicine	40	1,275
Honda S	Japan	Osaka City University Graduate School of Medicine	38	886
Lee WK	South Korea	Nune Eye Center	36	1,763
Kim JW	South Korea	Kim's Eye Hospital	36	385

We also analyzed cooperation between investigators; the node size within a collaboration network indicates the strength of the connections between every author. N. Yoshimura has close cooperation to other researchers according to the networks, as dose Cheung Chui Ming Gemmy. These researchers lead large-scale research teams and maintain close connections to others in the field of PCV research (Supplementary Figure 2).

### Analysis of the Keywords and Focuses of PCV Research

**Figure 4:**
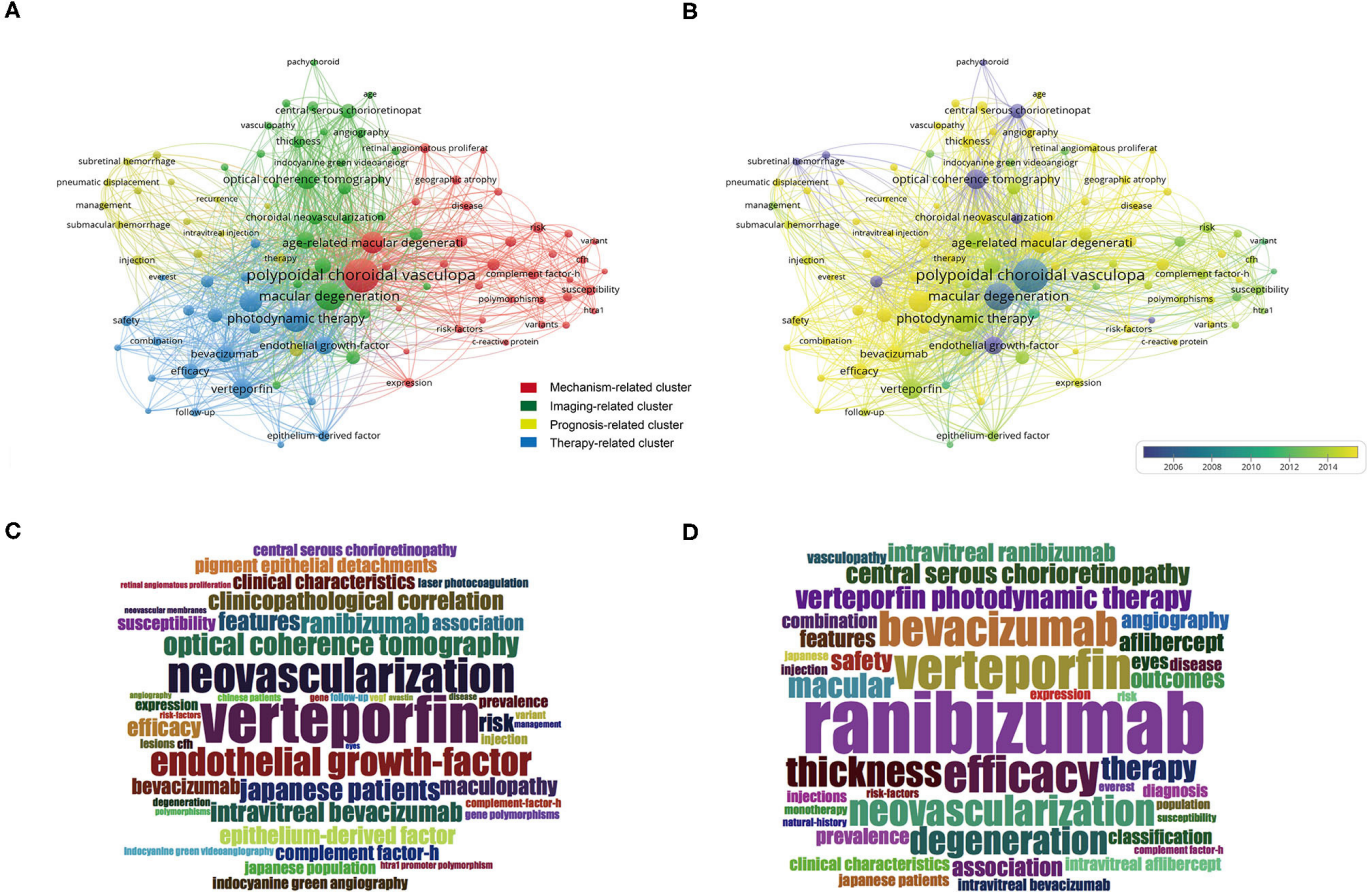
Keywords analysis by VOSviewer. **(A)** Keywords were divided into 4 clusters by co-occurrence frequency, including mechanism-related cluster (red), imaging-related cluster (green), prognosis-related cluster (yellow), and therapy-related cluster (blue). **(B)** All the keywords were color-coded by the average time of appearance. Yellow words appeared more recently, blue words appeared earlier. **(C)** The wordcloud map of the most frequent keywords before 2015. **(D)** The wordcloud map of the most frequent keywords since 2015.

Keywords analysis defined the most frequently used words and their linkages within the field of PCV research. We analyzed the keywords that appeared over 20 times across the 1,190 publications by VOSviewer. Merging repeated words and excluding meaningless ones resulted in 101 total keywords that can be divided into four primary clusters by co-occurrence frequency (Figure 4A), namely the mechanism-related cluster, the imaging findings-related cluster, the therapy-related cluster, and the prognosis-related cluster.

We also color-coded the keywords by average time of appearance (Figure 4B). The yellow words, such as ranibizumab (avg. pub. year 2016), aflibercept (avg. pub. year 2017), monotherapy (avg. pub. year 2018), and combination therapy (avg. pub. year 2016), were the ones that appeared most recently.

Given the publishing of the LAPTOP study that confirmed the superiority of anti-VEGF monotherapy over PDT in 2014 (10, 11), we divided the publications into two groups according to publication year; one group was papers published before 2015 while the other was those published since 2015. The most frequent keyword before 2015 was vertepoifin (Figure 4C), but it has been ranibizumab since 2015 (Figure 4D). Except for anti-VEGF therapy, we found several keywords appears recently and frequently, including therapy (avg. pub. year 2017), complement factor-H (avg. pub. year 2013), angiography (avg. pub. year 2015) (Figures 4B,D). They perhaps reflecting the trends of hotspots and the transformation of treatment options.

## Discussion

This study defined the frontier researches and examined the study trends in PCV-related research. We have found the increasing focuses of PCV-related research. Japan have published the most publications (428 publications), the most citations (14,504 citations in total) and the highest H-index value (61). We also predicted future hotspots for the next few years through bibliometric analysis. Our keywords analysis was classified into four clusters that showed the hotspots in PCV research, including mechanism-related studies, imaging-related studies, prognosis-related studies, and therapy-related studies. The average years of appearance for particular keywords confirmed that PCV angiography and treatment was the primary focus of most recent studies. These results defined the comprehensive progress made in PCV research, which can then guide the orientation of future research.

It makes sense that four of the top five countries/regions in terms of contributions to PCV-related publications are in Asia as Asian patients have the highest prevalence of PCV (12), as estimated by cross-sectional studies. For example, Byeon et al. (17) reviewed 392 nAMD cases in Korea. They diagnosed 24.1% of the patients with PCV using ICGA; Coscas et al. (18) examined 99 Japanese and 94 French nAMD cases. Among them, there were 48 (48%) Japanese patients with PCV, but only 8 (9%) French patients with PCV. This higher prevalence of PCV could explain the dominant position of Asian countries/regions within PCV research as increased prevalence within a large population could result in more patients, larger sample sizes, and more clinical studies. Japan began its research on PCV earlier than other countries/regions and has maintained leading status in every regard over the last 20 years. It is worth noting that China has maintained its rapid growth in the field, even though it began its PCV research relatively late (post-2004); it has surpassed both South Korea and the USA in terms of publication numbers since 2016. However, the studies of non-Asian countries were important part in this field as well (Figure 1B).

Relative research interest (RRI), an index representing the global attention being paid to a given field, in PCV has maintained growth over the last 20 years (from 0.0001 to 0.0005%, Figure 1D); this figure is consistent with the rapid increase rate (52.5%; 61 publications in 2006, 93 publications in 2007) of publications after 2006 (Figure 1D). This increase is also tied to the appearance of a landmark therapy in ocular vascular disease, namely anti-VEGF therapy. The United States FDA approved the first anti-VEGF agent for nAMD treatment (including PCV) in 2004 (19, 20). Currently, the treatment options for PCV include laser photocoagulation, verteporfin PDT, anti-VEGF therapy, and combinations thereof (1). Laser photocoagulation is only suitable for extrafoveal and extramacular polyps and may result in scars and RPE atrophy (1, 21); these factors keep it from being a primary option for PCV treatment. Photodynamic therapy (PDT) was widely used before the advent of anti-VEGF (22) and was the first safe, effective therapy for subfoveal lesions; it brought a higher polyp regression rate and less damage to the retina as compared to laser photocoagulation (23). It also brought more stable visual acuity, though it had plenty of disadvantages as well, including poor long-term vision, high costs, damage to the choroid, and RPE (24). Anti-VEGF changed the PCV treatment situation entirely. A series of clinical trials reported better long-term vision outcomes for patients treated with anti-VEGFs (10, 25, 26). The keyword cloud map also illustrates this transformation as the central word in PCV research changed from verteporfin to ranibizumab after the publication of the LAPTOP study results in 2014 (Figures 4C,D). In sum, anti-VEGF therapy, represented by ranibizumab, bevacizumab, and aflibercept, is currently recommended as the first-line treatment for PCV (27).

Color-coded keywords demonstrated that PCV treatment options have been research hotspots in recent years. Aflibercept monotherapy, a relatively new medication that can bind to VEGF-A, VEGF-B, and placental growth factor (PIGF) and has a higher binding affinity than ranibizumab and bevacizumab (28), usually performs similarly to combination therapy (anti-VEGF + PDT) and offers better visual outcomes (11, 29, 30). However, many patients still suffer from an inadequate response to anti-VEGF and require monthly injections (31). The polyp regression rate for patients who have done aflibercept monotherapy in the form of a treat-and-extend regiment for 1 year was 68.6% (32), which is to say that almost one third of patients have incomplete responses to anti-VEGF treatment. The presence of polyps always leads to rupture and exudation, which can cause the enlargement of PED as well as massive hemorrhaging (33). In addition, about 40% of patients treated with anti-VEGF experience subretinal fibrosis, which could be the cause of the non-response to treatment and poor visual outcome (34). These problems require significant research on better therapy options for PCV management; further studies and longer observation periods are necessary, though we also expect that the new anti-VEGF medications, like brolucimab, faricimab, and IBI302, will improve the treatment of PCV. Furthermore, gene therapy (RGX-314, gene therapy vector carrying a coding sequence for a soluble anti-VEGF protein, phase 2 clinical trial, https://clinicaltrials.gov/ct2/show/NCT04832724?cond=RGX-314&draw=2&rank=2) may entirely revolutionize PCV and AMD treatment.

In the study of PCV, we were able to determine the authors and institutions that are most likely to guide future PCV research. Citation numbers and H-index results can partially reflect the impact of particular researchers (35, 36) and, according to our results, Japan has the most citations, the highest H-index, and the most publications. It is worth noting that, although China has the second largest number of publications, it has fewer citations and a lower H-index than the USA, which is ranked fourth in publication numbers. This may be due to the uneven development and science research capacity of Chinese hospitals as well as their incomplete follow-up system and electronic medical records; their research quality, however, will continue to improve because many scientists and clinicians have realized and are trying to solve these problems. Several RCTs of PCV have been registered on *clinicaltrails.gov* and more and more high quality RCTs are being planned by Chinese researchers in the near future.

Our study also indicated that *Retina, Graefes Arch Clin Exp Ophthalmol*, and *AJO* are the primary journals for PCV publications, meaning that future developments in PCV research are likely to be published in one of them.

The keywords analysis demonstrated the factors that PCV research currently emphasizes, namely the mechanism-related cluster, inflammation, complement factor H, and gene variants. The mechanism of PCV has intimate connections with inflammation. Histopathologic findings have shown that CD68 positive macrophages infiltrated around the vessel (37), indicating the important roles of immune cells in the genesis and development of PCV.

Additionally, the complement system, a crucial part of the innate immune system, has been widely reported to be associated with age-related macular issues (38). The complement products elevated in the aqueous humor of PCV and drusen-associated nAMD (39). Several genetic studies have revealed the highly significant associations between AMD and many complement factors, including complement factor H (*CFH*)(40), *CFB/C2* (complement factor B and complement component 2) (41), and *C3* (complement component 3) (42). About 75% of AMD patients in European and North American countries/regions have been reported to carry *CFH* and *CFB/C2* variants (43). The activated complement system aggravates inflammation, form membrane-attack complex (MAC), induces tissue damage and neovascularization (44). In recent years, anti-complement therapy combined with anti-VEGF has been practiced in many clinical trials (45). Two clinical trials, which are pegcetacoplan (selective C3 inhibitor) and avacincaptad pegol (C5 inhibitor), have reduced the 29 and 27.4% rate of geographic atrophy (GA) leison enlargement (46). We are looking forward to the researches of anti-complement therapy on PCV. It can be predicted that anti-complement therapy will be a hot topic in the next few years.

Angiography is another keyword in recent years. The golden standard of PCV diagnosis relies on ICGA, single or multiple polyps can be identified at early phase of ICGA (11). It is an invasive examination with the injection of contrast agent, which can not be performed frequently. Therefore, a new consensus of PCV diagnostic criteria based on OCT was formulated and published in 2021 and try to improve the relevance ration of PCV through a fast, non-invasive method (9). The classical sign of PCV on OCT includes PED, double-layer sign, thickened choroid. The new consensus has raised 3 major criteria including sub-RPE ring-like lesion, en face OCT complex RPE elevation, and sharp-peaked PED. These criteria may be helpful for screening PCV in large populations, though they require more clinical practice to verify their efficacy. In addition, OCTA as a comparable non-invasive imaging technology by detecting blood flow (47, 48), has been widely used for diagnosis and follow-up visit. It has higher resolution, and can provide three-dimensional information and quantification of vessels (49, 50). OCTA could be one of the future trends in PCV angiography.

Our study has some limitations; we did not include papers that were not published in English, which may bias the results. Moreover, the most recent high-quality papers could not, for the most part, be cited at the time of our study, which may partially affect the results.

In conclusion, this study examined the published research on PCV and comprehensively analyzed the publication trends of the past 20 years. These results can help clinicians understand the history of PCV research, make better clinical decisions, and both predict and guide the future research orientation on PCV.

## Methods

### Search Methods

Web of Science Core Collection (WOSCC) was recognized as the most suitable database for bibliometric analysis. All the literatures included were published from 2001 to 2020. All the searches were conducted in 2021.11.22. The keyword was “TS = polypoidal choroidal vasculopathy”, including all the publications with keywords in titles, abstracts, author keywords.

**Figure 5:**
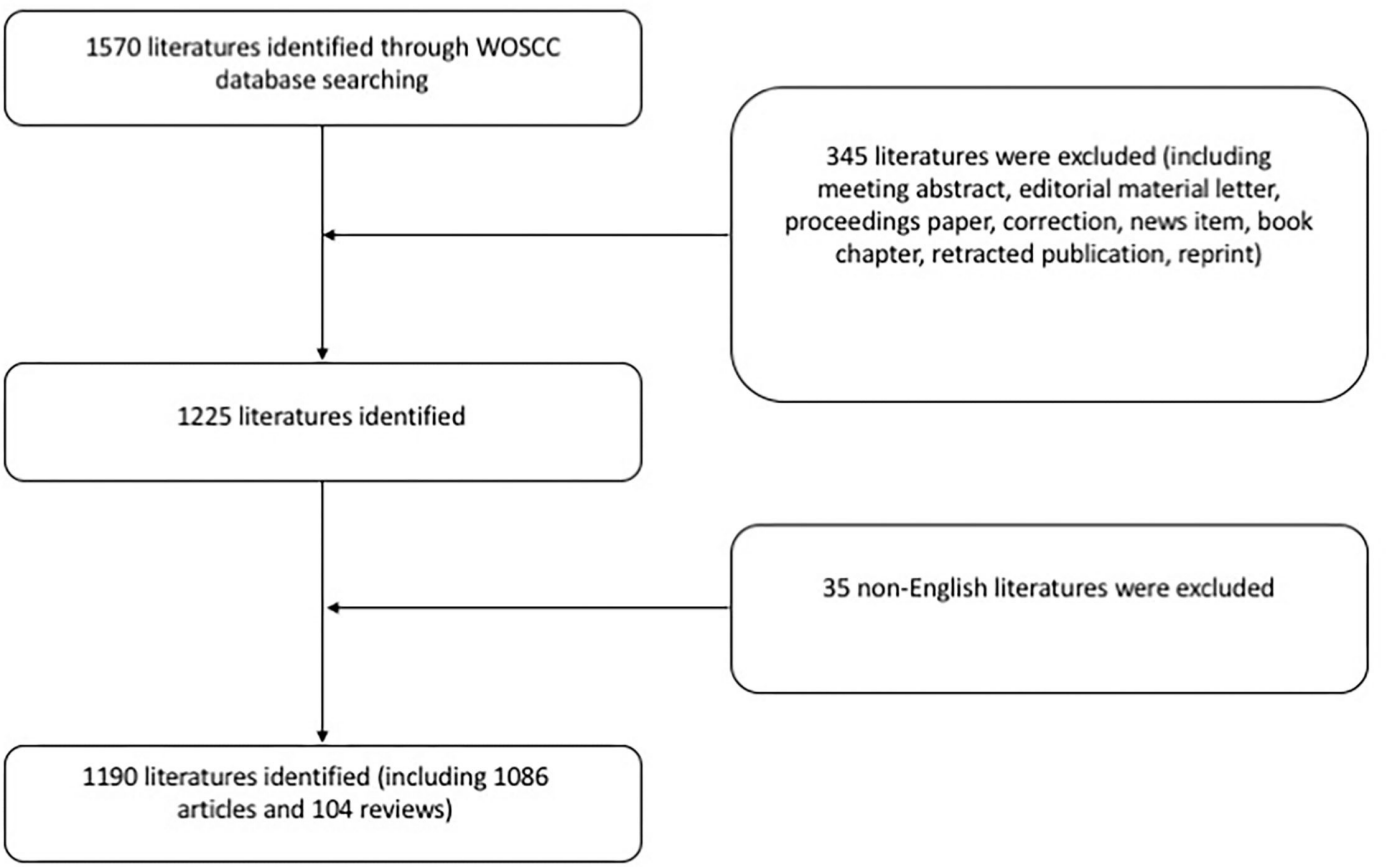
Flow chart of the search strategy of our study. Only English articles and reviews were reserved.

1,570 literatures were identified through WOSCC database searching. 345 publications were excluded according to the document type (articles and reviews were kept). 35 non-English papers were excluded. Finally, 1,190 literatures were identified including 1,086 articles and 104 reviews (Figure 5).

### Data Collection

All the data were extracted and downloaded from WOS databases (i.e., publication numbers, countries and regions, authors, citations, and H-indexes). Microsoft Excel 2010, VOSviewer, a software of which can construct and visualize bibliometric networks, (Leiden University, Leiden, the Netherlands),GraphPad Prism 8.0.0 (131), R (R. app GUI 1.70) and Rstudio (1.2.1335) were used to input and analyze data.

### Bibliometric Analysis

The descriptive indexes were exacted from WOS and calculated by Microsoft Excel 2010. Relative research interest (RRI) represents the global attention in this field, which was measured as the number of publications in this field divided by all publications of all fields. H-index can partially reflect the impact of researchers, collected from WOS database. The prediction model is *f* (*x*) = *ax*^3^ + *bx*^2^ + *cx* + *d*, which was the matched curve generated from the cumulative publications. The co-occurrence network of keywords was constructed from titles and abstracts by VOSviewer. Frequencies over 20 was the criteria of the exhibited keywords. Average appearing year (AAY) was used to assess the novelty of the keywords.

## Data Availability Statement

The raw data supporting the conclusions of this article will be made available by the authors, without undue reservation.

## Author Contributions

All authors listed have made a substantial, direct, and intellectual contribution to the work and approved it for publication.

## Funding

This study was supported by the National Natural Science Foundation of China (81730026, 82171076, 8210041148) National Science and Technology Major Project (2017YFA0105301), Science and Technology Commission of Shanghai Municipality (20Z11900400) supported by Shanghai Hospital Development Center (SHDC2020CR2040B, SHDC2020CR5014).

## Conflict of Interest

The authors declare that the research was conducted in the absence of any commercial or financial relationships that could be construed as a potential conflict of interest.

## Publisher's Note

All claims expressed in this article are solely those of the authors and do not necessarily represent those of their affiliated organizations, or those of the publisher, the editors and the reviewers. Any product that may be evaluated in this article, or claim that may be made by its manufacturer, is not guaranteed or endorsed by the publisher.

## Supplementary Material

Supplementary Figure 1 The co-occurrence of 22 countries/regions, showed the cooperation among countries/regions.

Supplementary Figure 2 The co-occurrence of PCV scholars, showed the cooperation among researchers.

Supplementary Figure 3 **(A–D)** The heatmap of each group. Every row in the heatmap represents a publication, the x axis means year, and the color represents the total citation number. **(D)** The distribution of publication year of each group.
