# Supplementary figures and images for: Publication Trends of Research on Polypoidal Choroidal Vasculopathy During 2001–2020: A 20-Year Bibliometric Study
Source: Front Med (Lausanne). 2022 Jan 31;8:785126. doi: 10.3389/fmed.2021.785126 (PMC8841421; doi:10.3389/fmed.2021.785126)

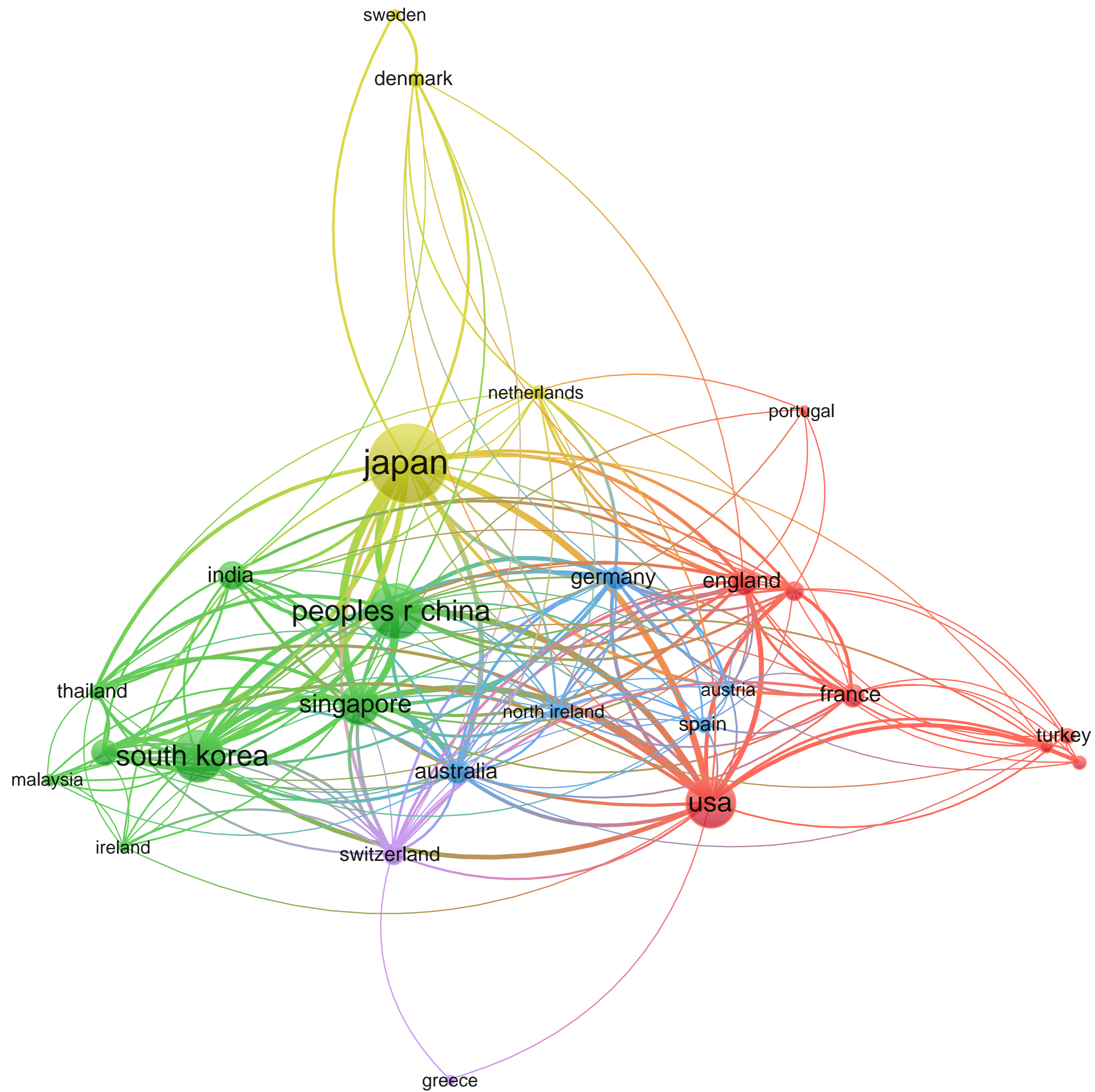

Supplement: Supplementary Figure 1 — The co-occurrence of 22 countries/regions, showed the cooperation among countries/regions. [file Data_Sheet_1.PDF]

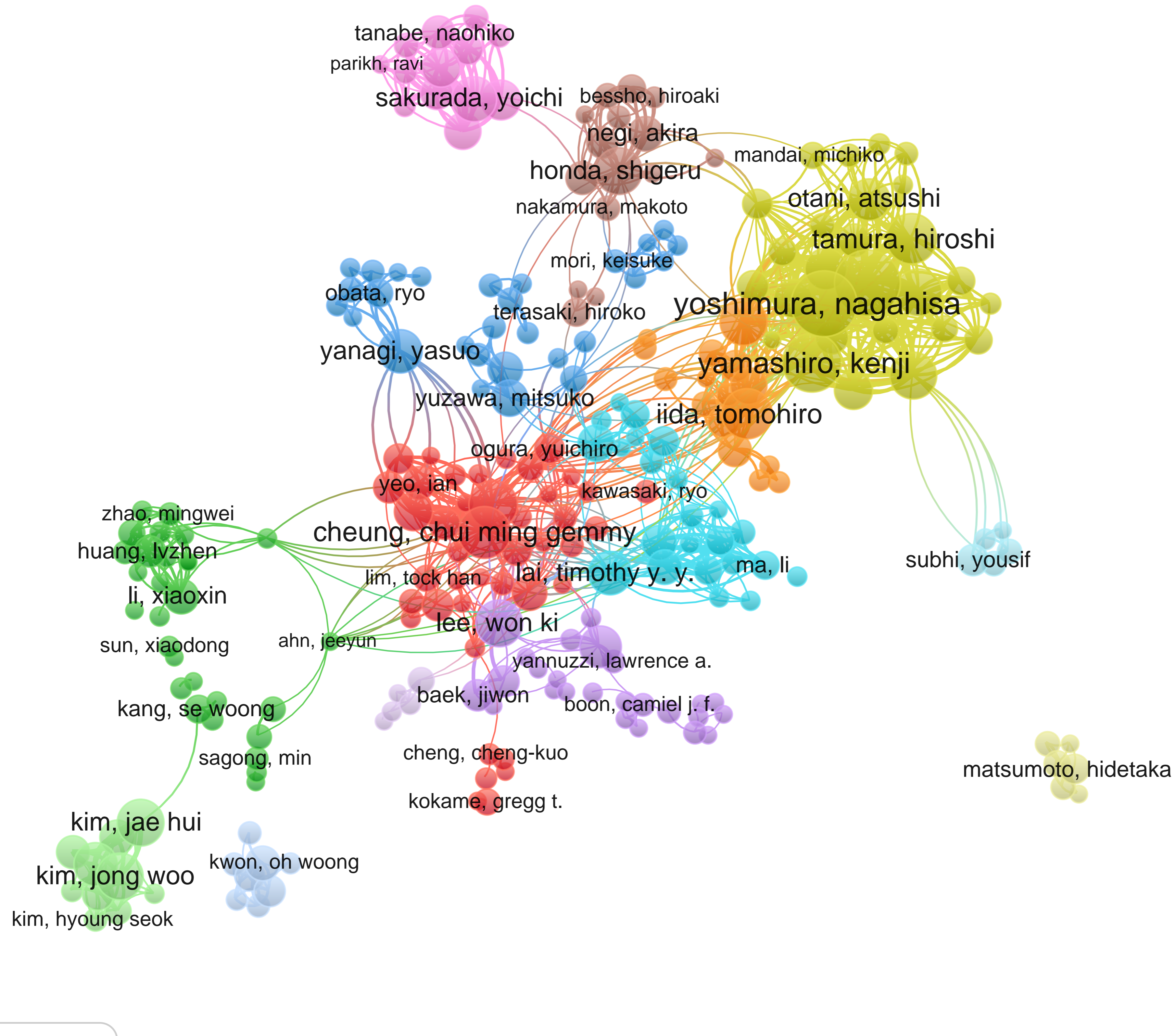

Supplement: Supplementary Figure 2 — The co-occurrence of PCV scholars, showed the cooperation among researchers. [file Data_Sheet_2.PDF]

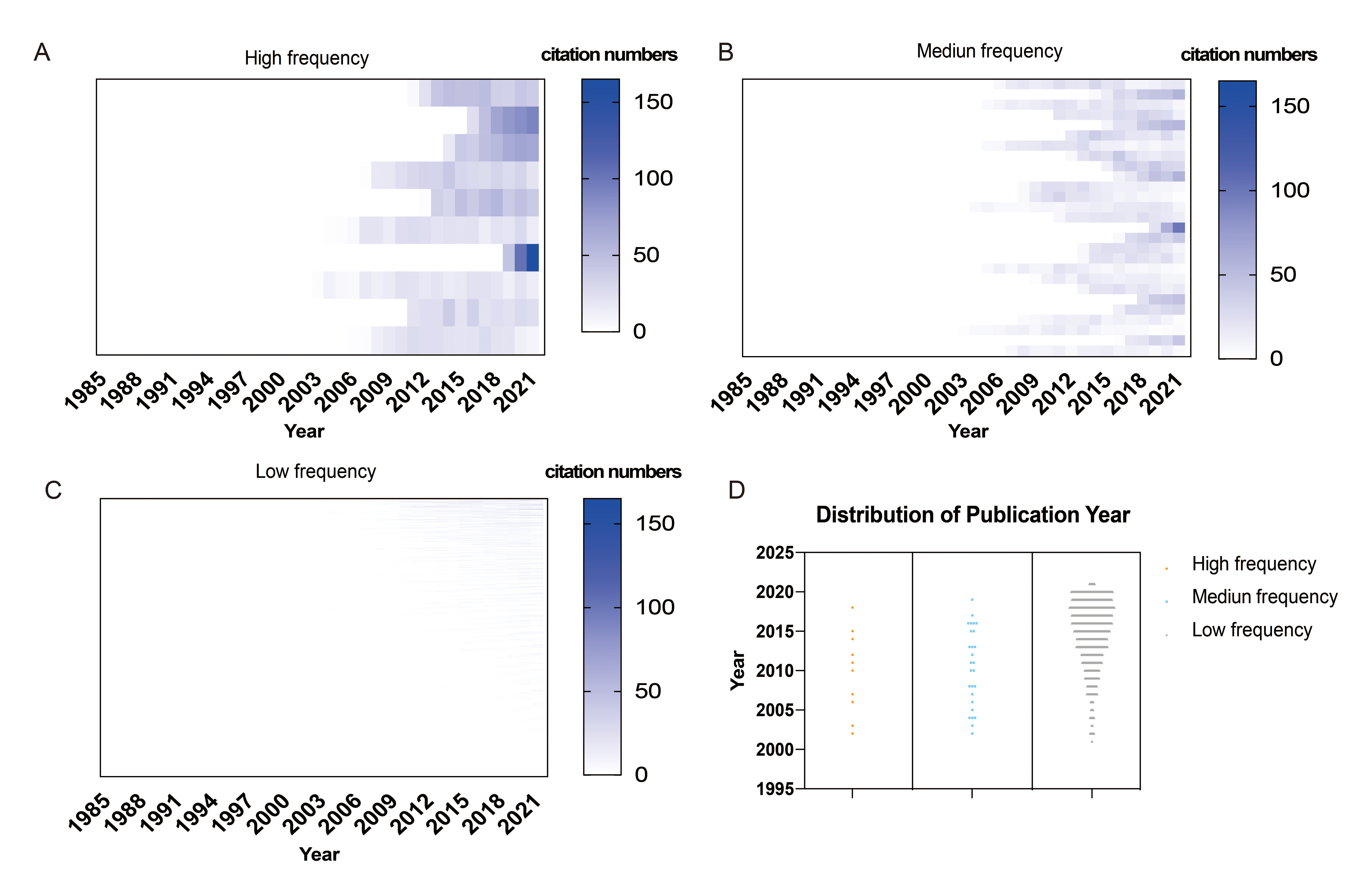

Supplement: Supplementary Figure 3 — (A–D) The heatmap of each group. Every row in the heatmap represents a publication, the x axis means year, and the color represents the total citation number. (D) The distribution of publication year of each group. [file Image_1.JPEG]
